# A complex systems approach to obesity: a transdisciplinary framework for action

**DOI:** 10.1177/17579139231180761

**Published:** 2023-07-03

**Authors:** C Griffiths, D Radley, P Gately, J South, G Sanders, MA Morris, K Clare, A Martin, A Heppenstall, M McCann, J Rodgers, J Nobles, A Coggins, N Cooper, C Cooke, MS Gilthorpe, L Ells

**Affiliations:** Obesity Institute, School of Sport, Leeds Beckett University, Headingly Campus, Leeds LS6 3QS, Yorkshire, UK; Obesity Institute, School of Sport, Leeds Beckett University, Leeds, UK; Obesity Institute, School of Sport, Leeds Beckett University, Leeds, UK; Centre for Health Promotion Research, School of health, Leeds Beckett University, UK; Obesity Institute, School of Sport, Leeds Beckett University, Leeds, UK; Leeds Institute for Data Analytics and Leeds Institute for Medical Research, University of Leeds, Leeds, UK; Obesity Institute, School of Health, Leeds Beckett University, Leeds, UK; Leeds Institute of Health Sciences, School of Medicine, University of Leeds, Leeds, UK; School of Political and Social Sciences, MRC/CSO Social and Public Health Sciences Unit, University of Glasgow, Glasgow, UK; MRC/CSO Social and Public Health Sciences Unit, University of Glasgow, Glasgow, UK; International Business School, Teesside University, Middlesbrough, UK; Obesity Institute, School of Health, Leeds Beckett University, Leeds, UK; Essex County Council, Chelmsford, UK; Suffolk County Council, Ipswich, UK; Obesity Institute, School of Sport, Leeds Beckett University, UK; Obesity Institute, School of Sport, Leeds Beckett University, Leeds, UK; Obesity Institute, School of Health, Leeds Beckett University, Leeds, UK

*In this article, Dr Claire Griffiths et al. present a simple framework of a complex problem, which provides all stakeholders with the foundation to implement a systems approach to obesity. It demonstrates the need for transdisciplinary working to ensure the individual, local, national and international perspectives are considered*.

**Table:** Study Group Details.

Complex Obesity Systems Study Group		
Forename	Surname	Institution
Karen	McCormack	Suffolk County Council
Dorothy	Monekosso	Durham University
Alexandra	Potts	Leeds Beckett University
John	Preston	Leeds Beckett University
Colette	Brolly	Public Health Agency, Gransha Park House, Gransha Park, Clooney Road, L, DERRY
Jenny	Thompson	North Yorkshire Council
Jim	McManus	Association for the Directors of Public Health, UK.
Katie	Shearn	Sheffield Hallam University
Robert	Copeland	Sheffield Hallam University
Oliver	Mytton	Great Ormond Street. Institute of Child Health, University College London.
Jutka	Halberstadt	Department of Health Sciences, Faculty of Sciences, Vrije Universiteit Amsterdam, Amsterdam Public Health research institute, Amsterdam, The Netherlands
Sandra	James	Office for Health Improvement and Disparities, East of England Region
Hannah	Sharpe	Leeds Beckett University
Clare	Jackson	Obesity Voices, Leeds Beckett University
David	Tumilty	Public Health Agency, Gransha Park House, Gransha Park, Clooney Road, L, DERRY
Paul	Ogden	Local Government Association, UK.
Ruth	Everson	North Yorkshire Council
Sophia	Bird	Public Health Wales, Health Improvement Division, No 2 Capital Quarter, Tyndall Street, Cardiff
Tim	Fielding	Leeds City Council
Anastasios N.	Delopoulos	Aristotle University of Thessaloniki, Greece.
Ana	Rito	National Institute of Health Dr. Ricardo Jorge, Portugal
Helen	Ingle	North Yorkshire Council
Jaap	Seidell	Vrije Universiteit Amsterdam, Amsterdam Public Health research institute, Amsterdam, The Netherlands

Obesity is a major public health challenge which continues to increase and disproportionally affects vulnerable population groups, resulting in widening health inequalities.^1^ There is consequently an urgent need for innovative approaches to identify and implement evidence-based policy and practice to prevent and treat obesity which has been accelerated by the COVID-19 pandemic.^2^

**Figure 2.**
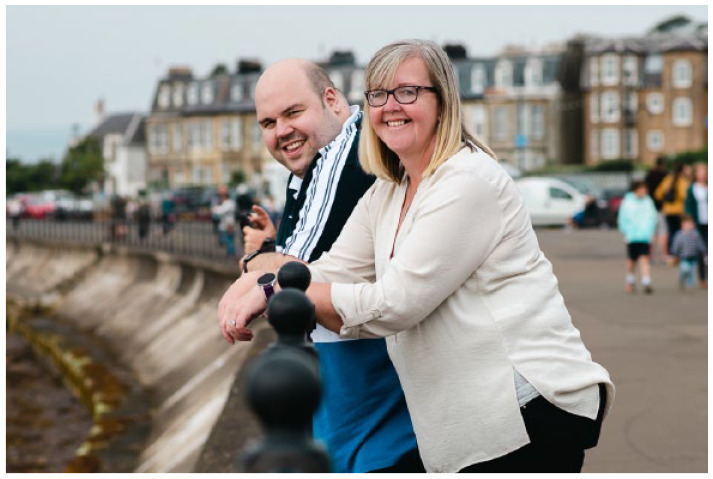
credit: ecpomedia.org

The population levels of obesity are driven by numerous interacting political, economic, environmental, social, cultural, digital, behavioural, and biological determinants. However, causal links between determinants and how they vary between different groups of individuals are not well defined. The identification, implementation, and evaluation of effective responses to the prevention and treatment of obesity require a set of approaches that work within this complexity.^3,4^

The limited efforts to date reflect a misunderstanding of the nature of the chronic and complex nature of obesity, and importantly, a limited understanding of how the multifaceted nature of the problem should influence how research, policy, and practice approach it. To date, the evidence underpinning the current approach does not reflect the complexity of the condition:

- Evidence is largely generated by tools and methods developed to answer questions about the effectiveness of isolated interventions, commonly grounded in linear models of cause and effect. This is the pathway between a cause, for example, exposure to fast food restaurants, and the outcome, obesity, is assumed to be linear, when it is far more complex than this.
- There is a focus on individual behaviour, yet social and structural determinants of health have a far greater influence on obesity and contribute more to health inequalities.^1^ It is acknowledged that we live in an obesogenic environment,^5^ yet most approaches to addressing obesity are focused on behaviour change to support individuals adopt healthy weight behaviours, with little (or no) consideration of the environment in which they live.^4^
- Outcomes are largely measured in the short term and the effects of efforts to reduce population obesity will take many years to be realised.
- Effectiveness is primarily determined by a narrow focus on weight change, which fails to capture the underlying complexity. Instead of investigating whether a single intervention is (cost-)effective in terms of fixing the problem (i.e. obesity), we need to understand how actions drive positive changes within the system.

A systems approach captures and responds to complexity through a dynamic way of working: bringing together academic, policy, practice, and community representatives to develop a ‘shared understanding of the challenge’ and to integrate action to bring about sustainable, long-term systems change.^3,4^ The benefit of a systems approach to addressing population levels of obesity has been outlined: in 2013, the EPODE logic model^6^ retrospectively provided insight into the system dynamics of the programme; the ‘Improving the Health of the Public by 2040’ report^3^ acknowledged that responses to major public health challenges require a wider set of approaches; in 2017, Rutter *et al.*^4^ called for ‘a complex systems model of evidence for public health’, which was echoed in 2019, as part of *The Lancet* commission on obesity.^7^ More recently, the logic model underpinning the Amsterdam Healthy Weight Approach (AHWA) was published.^8^ There are also examples of projects that have embraced system approaches in an applied setting,^9–11^ as well as toolkits,^12^ guidance documents,^13–15^ and operational frameworks.^16–19^ These resources demonstrate that the concept of a systems approach to obesity is not new, and importantly that systems methods do not have to replace traditional methods, but instead incorporate and enhance them.^20,21^ Despite this activity and rhetoric, systems approaches are rarely operationalised in ways that generate relevant evidence or effective policies.

## A Transdisciplinary Complex System Framework for Obesity

**Figure 1.**
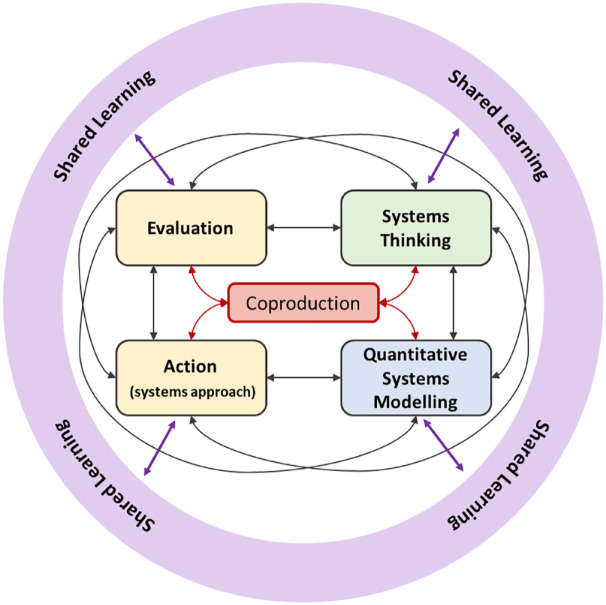
Complex systems framework for obesity.

The ‘Improving the Health of the Public by 2040’ report^3^ highlighted the importance of transdisciplinary research to establish a robust understanding of the long-term impacts of many of the wider drivers of public health that cut across local, national, and global environments. We developed a transdisciplinary consortium, representing multidisciplinary academics, policy, practice, and community representatives, as well as individuals with lived experience (see study group details), to coproduce a complex systems framework for obesity (Figure 1). This framework brings together six concepts: systems thinking, quantitative systems modelling, action (systems approach), evaluation, shared learning, and at its core, coproduction to design, implement, and evaluate an approach to obesity which is consistent with the underlying complexity. Although arranged sequentially in a clockwise fashion, the concepts need not be implemented sequentially and can be repeated as necessary to support ongoing development. Each distinct concept could be considered in isolation; indeed, the current evidence base for systems approaches to obesity management and prevention is dominated by research with a ‘system thinking’ lens^20,21^ and, although it is not necessarily wrong to consider these ‘concepts’ in isolation, it is important to understand how they fit together to drive system change. The value of blending multiple methods from the systems toolkit (rather than driving the research with a single tool as the lens) has been illustrated by the Childhood Obesity Modelling for Prevention and Community Transformation (COMPACT) team.^22^ It is the synergy of the different concepts to truly capture the complexity that makes this framework innovative and ambitious.

*Coproduction* is at the heart of our systems approach, to ensure it is built around the needs, experiences, and knowledge of academics, policy makers, practitioners, organisations, and community members. Stakeholder views may differ regarding the nature of the problem, appropriate strategies for addressing the problem, or how to implement those strategies. Although there is consensus from public health experts on how to address population obesity,^7^ the multiple perspectives of stakeholders, which are a symptom of the complexity, challenge this consensus.

*Systems thinking* (qualitative system modelling) is concerned with the structure of a system and is underpinned by three core principles:^16,23^ first, defining *system boundaries*, determining what is considered in or out of the system and how the system will be conceptualised vis-à-vis its external environment. Boundaries that are set too widely may overwhelm action or evaluation; boundaries set too narrowly exclude important system perspectives and partners. Second, we must make sense of the *inter-relationships* between parts of the system. Relationships include the formal and informal connections, exchanges, or interdependencies among system parts, whether they are professional partnerships, social relationships, collaborative networks, communications channels, funding streams, flows of information, data or knowledge. Third, it is important to view the system from multiple *perspectives*; system stakeholders will have different perspectives or pursue different agendas in a particular situation, which reiterates the importance of coproduction. System thinking methods used in obesity research may include group model building (GMB) and qualitative system mapping (QSM)^21,24^ to facilitate stakeholders and evaluators in restructuring their individual and collective understanding of the system in question.

*Quantitative systems modelling* allows the characteristics of complex systems to be captured and embedded in quantitative models, to understand how interconnections among the various individuals and organisations give rise to emergent and dynamic behaviours or properties. System modelling methods used in obesity research include system dynamics modelling, (social-) network analysis, and agent-based modelling.^20^ The aim of such models is not to replicate the ‘real world’ precisely, but rather to create a helpful abstraction to evaluate potential changes and the mechanisms that drive them. It is important that any quantitative systems modelling is informed by, and built upon, the insight gathered from system thinking methods, thus accounting for the multiple perspectives of various stakeholders. The evolution and utilisation of quantitative systems modelling aligned to outputs from systems thinking methods have been used to describe how system stakeholders use their social networks to diffuse knowledge about and engage with childhood obesity prevention efforts.^22^

*Action (systems approach)* needs to follow. Few system approaches demonstrate informed action in a real-world setting and no approach is informed by blending multiple methods from the systems toolkit (although many ‘system approaches’ have used components in isolation).^25^ Although both system thinking methods and quantitative systems modelling pursue a process to create a systemic awareness of a problem situation, and their methods may (or may not) shed light on the same systemic elements, their merger is what provides the most comprehensive understanding of system functioning. For example, actions developed based only on the outcomes of systems modelling without a multiperspective understanding of the system (i.e. systems thinking) may not be practically implementable and might be viewed as flawed by stakeholders. Conversely, a system thinking approach that qualitatively describes the system with no formal modelling is likely to overlook key uncertainties and system behaviours that a quantitative modelling approach could identify. Fundamental to the *action of a systems approach* is understanding the different perspectives of stakeholders on what constitutes ‘evidence’ and what value different stakeholders place on ‘evidence’. Action in practice is informed by a complex and dynamic range of factors beyond simply the robustness of the methods/strength of the evidence (e.g. political views and policies, vested interests, biases, public opinion, competing priorities).

*Evaluation* is essential. Although guidance on how to evaluate complex interventions, including complex interventions within complex social systems, has been published,^13–15,19,20,26^ they all call for new and innovative approaches to complex systems evaluation. System approaches are currently being used with limited knowledge of the likely effectiveness of any individual or collective action being taken.^25^ More recently though, the ENCOMPASS framework^17^ and the Scottish National evaluation protocol^27^ have been published to support researchers in designing systems evaluations. Within our framework, the evaluation captures the attribution (i.e. what proportion of the outcome was produced by the action) but also the contribution (i.e. how reasonable is it to believe that the action(s) and the behaviours of individuals contributed to system changes). The inherent complexity of a systems approach, where the route to change could be nonlinear and cannot easily be predicted beforehand, requires a flexible, adaptive, and iterative evaluation design. Rather than undertaking a static response to an intervention or action at fixed timepoints and with predetermined questions, a system evaluation needs to adjust in response to potentially important outcomes that emerge.

*Sharing learning* is central to the success, impact, and legacy of a systems approach. All stakeholders need to be able to access information and data that is meaningful and useful to them; they must see their place in the system and be aware as to how they are influenced by other factors in the system. The Academy of Medical Sciences report recommends that we should ‘harness new technology and the digital revolution’ requiring us to collectively address issues associated with data access, ethics, trust, regulation and skills.^3^ Furthermore, decisions in the ‘real world’ are often evidence-*informed* rather than evidence-*based*, and decisions are sometimes taken quickly and for a range of complex reasons. The ambition of shared learning within our framework goes beyond publishing scientific evidence (although this remains important). We must improve the knowledge base and enhance capacity within the field leading to improved decision- and policy-making and improved service delivery. The full societal value of a systems approach will not be realised until it is translated into improved health and health equity, and this will take considerable time. We must ensure that all stakeholders actively contribute to the outputs, rather than simply receiving them, thus enhancing the real-world applicability. This will require iterative and meaningful engagement with all sectors of society, including practitioners, policymakers, the commercial sector, and the public.^3^

## Summary

Our complex systems framework (Figure 1) complements and extends existing international best practise by extending methodologies in the design, implementation, and evaluation of obesity actions. Perhaps most importantly, this is the first framework to be coproduced by a transdisciplinary team with a holistic understanding of the wide range of obesity determinants, and the skills and approaches necessary to address them (see study group details). The aim is that this simple framework, of a complex problem, will provide stakeholders with the foundation to implement a systems approach to obesity. To achieve this, research funding, research activity, and the evidence base need to rebalance the distribution of projects that take a complex system approach.^4^
